# Biosecurity Policy in the US: A Critical Assessment

**DOI:** 10.3389/fpubh.2014.00110

**Published:** 2014-08-04

**Authors:** Ori Lev, Limor Samimian-Darash

**Affiliations:** ^1^Department of Public Policy and Administration and Masters Program in Public Policy, Sapir College, D.N. Hof Ashkelon, Israel; ^2^The Federmann School of Public Policy and Government, The Hebrew University of Jerusalem, Jerusalem, Israel

**Keywords:** dual use research of concern, risk-benefit assessment, biosecurity policy, H5N1, US Government Policy

This commentary will critically evaluate the *US Government Policy for Oversight of Life Sciences Dual Use Research of Concern* with a special focus on the process of assessing the risks and benefits of studies that are deemed to be dual use research of concern (DURC). Assessing the risks and benefits of DURC studies is probably the most complicated part in implementing the policy. Curiously, little attention has been paid to this complex process. This paper details how this process is conducted and points out a major challenge it faces. We will suggest that this challenge is difficult to resolve thereby requiring further policy development.

On March 29, 2012, the US Government issued *The United States Government Policy for Oversight of Life Sciences Dual Use Research of Concern* (1). The policy was published after months of controversy over the issue of whether studies that enhanced the transmissibility of the highly pathogenic avian influenza (HPAI) H5N1 viruses should be published and if so in what form (2). The main concern these studies have generated was that if they are to be published in full malevolent actors might misuse the information included in them to construct a deadly virus. Issuing the policy was, at least in part, a way for the government to demonstrate that it is taking control of the events and is pursuing steps that would mitigate some of the concerns that were raised about these studies.

Importantly, the policy gave the US Government tools; it lacked when the H5N1 controversy erupted. Examples of such tools are listed below.

The new policy, for example, provides the government with the authority to terminate funding of research that is deemed too risky (1). This is an extreme measure that is unlikely to be used; however, including it in the policy reveals not only the sense of pressure government officials felt given the circumstances, but also their belief that the government should have a very wide scope of tools that could be employed to govern this research. Other tools the policy provides are related to determining the biosafety conditions under which the research is done and a periodic assessment of the research for its potential to be DURC. This periodic assessment is a direct result of the H5N1 controversy, in which it seemed the US Government was caught by surprise by the ensuing crisis. The periodic review allows the government to be constantly updated on the state of the research portfolio it funds. These steps are crucial given the potential that more DURC studies are likely to be conducted.

To decide which of these steps should be applied the policy articulates a four-step process (1). The first step is to determine whether the research involves a pathogen from a list of 15 infectious agents and toxins that are deemed most lethal. The second step is to determine whether that research performs an experiment that falls under any of the seven categories of experiments listed in the policy.

If the study meets these two criteria, a third step is pursued, specifically, determining whether the study meets the DURC definition set out in the policy. The definition is as follows:

“DURC is life sciences research that, based on current understanding, can be reasonably anticipated to provide knowledge, information, products, or technologies that could be directly misapplied to pose a significant threat with broad potential consequences to public health and safety, agricultural crops and other plants, animals, the environment, materiel, or national security.”

This definition was adopted with a few revisions from an earlier definition that the National Science Advisory Board for Biosecurity has articulated in its report “Proposed Framework for the Oversight of Dual Use Life Sciences Research: Strategies for Minimizing the Potential Misuse of Research Information” (3):

“Research that, based on current understanding, can be reasonably anticipated to provide knowledge, products, or technologies that could be directly misapplied by others to pose a threat to public health and safety, agricultural crops and other plants, animals, the environment, or materiel.”

The definitions are similar but have two differences worth pointing out.

The first difference is that the phrase “by others” was eliminated from the new DURC definition. This phrase was originally intended to express the idea that scientists are well-intended when conducting research, while “others,” malevolent actors, might misapply their research. Eliminating the phrase “by others” could be understood as suggesting that scientists themselves could misapply research findings. This is probably the result of the 2001 U.S. Anthrax attacks, which were allegedly undertaken by a scientist and resulted in 5 deaths and 17 injuries (4). Another possible reason for this change is the acknowledgment that scientists might accidentally misapply their research.

The second difference is the addition of the phrase “a significant threat with broad potential consequences” to the new definition. This addition is intended to help those that assess particular studies; it aims to provide them with a more specific criterion with which they can assess the risks of misuse. However, this addition though intended to help is still vague and is likely to be interpreted in an inconsistent way thereby endangering the effectiveness of the policy. This vagueness, we propose, should be addressed. One way in which this could be achieved is by entirely eliminating the third step and moving directly to the fourth step, which calls for a robust risk benefit assessment. This assessment is the only way in which the magnitude of the risks and their likelihood will be determined; the third step is redundant and confuses the process.

As said, the fourth step of the policy calls for an assessment of the risks and benefits of the studies that were determined to be DURC. The risk benefit assessment is utilized to decide whether any of the tools the policy provides ought to be used: should the study design be modified, should it be done under different conditions, should its publication be subject to any limitations, should its funding be terminated?

However, the fourth step presents a serious challenge. A challenge that we would argue ought to be seriously considered and addressed if possible. To be clear, this challenge is related to the policy as it is currently set out. The policy places the responsibility for conducting the risk benefit assessment in the hands of scientists; however, generally speaking scientists lack the knowledge and capabilities required for assessing the risks of misuse.

A risk benefit assessment for DURC is unique in its focus on the risks of misuse by malevolent actors (5). In other words, a DURC assessment is essentially a biosecurity assessment. Yet, the scientific community is not equipped with the knowledge, expertise, and capabilities to conduct a security assessment (6).

The scientific community is well placed to assess the public health benefits of their research. They can provide sound assessments of the likelihood of the benefits and their magnitude. We would also argue that they are well placed to assess the magnitude of the harms if the research is misused. They might even be able to provide a sound assessment of the feasibility of misusing the information. That is, they are able to attest to the technical abilities needed and whether they are easy or difficult to acquire.

Yet, scientists are incapable of assessing the likelihood that a given study would be misused; they do not have access to such information. This kind of information is not publically available. In particular, they have no way of knowing if there is any group with the intention of misusing the research information or materiel. They also lack any information regarding the capabilities of any group that might have the intention to misuse research findings. Moreover, they do not have access to knowledge about efforts to prevent groups who intend on doing harm and the success of such efforts. Without this information, the scientific community cannot assess DURC.

The kind of information that is needed for a comprehensive assessment would only exist within the security and intelligence community. This kind of information is sensitive and thus it is unlikely that it will be shared with the scientific community unless a reliable mechanism to convey such information is established.

However, one might only imagine the difficulties of establishing such a mechanism. Scientists would have to get security clearances; they would also have to be trained on how to interpret such information reliably (6). This has been done to a limited extent through the creation of the Biological Sciences Experts Group (BSEG) in which a limited number of scientists and science administrators receive clearance and are briefed from time to time (7). Yet this model cannot meet the demands of the new policy as it is too limited in scope and authority. The security and intelligence communities, it is safe to say, are unlikely to agree to extend this type of mechanism. They would object to sharing sensitive and classified information with a growing number of people outside their institutions. The risks of such a mechanism are too high.

One might suggest a middle way in which security personnel would participate in the assessment process and provide input on whether a given study has high or low likelihood to be misused. But even this middle way would be problematic as scientists would probably demand greater transparency if they are to accept any limitations on their freedom to pursue scientific inquiries. Greater transparency, however, is unlikely to be forthcoming as providing more detailed information could have detrimental effects to the intelligence operations.

This divides between the interests of the scientific community and the security and intelligence communities must be bridged if we are to address the DURC challenge effectively. Leaving the policy as it currently stands seems unsustainable. This is because it would lead to problematic outcomes. Without information on the likelihood of misuse scientists would have to turn to “educated guesses,” under such conditions they are likely to make two kinds of mistakes. First, they might place low likelihood of misuse on studies that have a high chance to be misused, thereby endangering national security. Second, they might curtail important research on the grounds that it encapsulates high risks of misuse although in reality such research is unlikely to be misused thereby harming important advances that could benefit public health.

As suggested, to avoid these potential mistakes a way for the security establishment and the scientific community to collaborate must be sought. If such a mechanism is impossible to set up, policy makers must convey to the public that the DURC policy has limits. Moreover, scientists conducting DURC reviews must be aware that their determinations are subject to the kinds of mistakes that we pointed out. Should they then err on the side of caution or not is a difficult question that should receive close scrutiny (8, 9).

It is important to note that there are still policy tools available to the government if the dangers of misuse are increasing. It is the responsibility of the security establishment to constantly be on the lookout for malevolent actors who intend to misuse scientific information. If these risks are increasing dramatically they could demand that certain lines of research be done in a classified way. The scientific community in the US as well as in other countries is unlikely to easily endorse this approach, yet it did so already when it became clear that openly conducted nuclear physics research poses severe risks to society (10). This is not the situation we are facing with regard to life sciences research. Yet, it is important to realize that the limitations of the DURC policy can be addressed in an alternative way if warranted.

To conclude, the US Government policy for the oversight of DURC is an important step in attempting to balance the need for scientific progress and safeguarding our societies. Yet the policy is formulated in such a way that the risks of misuse cannot be accurately assessed. To fulfill its goal further policy development efforts are necessary.

## Conflict of Interest Statement

The authors declare that the research was conducted in the absence of any commercial or financial relationships that could be construed as a potential conflict of interest.
