# Corrigendum: Biosecurity Policy in the US: A Critical Assessment

**DOI:** 10.3389/fpubh.2015.00247

**Published:** 2015-11-03

**Authors:** Ori Lev, Limor Samimian-Darash

**Affiliations:** ^1^Department of Public Policy and Administration and Masters Program in Public Policy, Sapir College, D.N. Hof Ashkelon, Israel; ^2^The Federmann School of Public Policy and Government, The Hebrew University of Jerusalem, Jerusalem, Israel

**Keywords:** dual use research of concern, risk-benefit assessment, biosecurity policy, H5N1, US Government Policy

The authors would like to add the following correction to the Opinion article to now include the acknowledgment of funding for their research.

## Conflict of Interest Statement

The authors declare that the research was conducted in the absence of any commercial or financial relationships that could be construed as a potential conflict of interest.

